# Homogeneity of Memory Errors in Abstract Visual Pattern Recall

**DOI:** 10.5964/ejop.v15i3.1685

**Published:** 2019-09-27

**Authors:** Stephen P. Badham, Christopher Atkin, Antonio Castro

**Affiliations:** a Department of Psychology, Nottingham Trent University, Nottingham, UK; University of Bari "A. Moro", Bari, Italy

**Keywords:** visual matrix patterns, iterated learning, serial reproduction, inductive bias, memory reconstruction

## Abstract

In memory tests, recalled information can be distorted by errors in memory and these distortions can be more memorable than the original stimuli to a later learner. This is typically observed over several generations of learners but there is less exploration of the initial distortions from the first generation of learners. In this article, participants studied visual matrix patterns which were either erroneous recall attempts from previous participants or were random patterns. Experiment 1 showed some evidence that material based on previous participants’ recall data was more memorable than random material, but this did not replicate in Experiment 2. Of greater interest in the current data were homogeneity in the memory errors made by participants which demonstrated systematic recall biases in a single generation of learners. Unlike studies utilising multiple generations of learners, the currently observed distortions cannot be attributed to survival-of-the-fittest mechanisms where biases are driven by encoding effects.

It is well established that human memory is not just an objective, unbiased storage system that records all incoming sensory information. Amongst other factors, it can be influenced by the direction of attention (e.g., Mulligan, 1998), by the amount of processing applied to stimuli (Craik & Lockhart, 1972), and by an individual’s familiarity with study material (Chase & Simon, 1973; Miller, 2003). The last of these factors is linked to the notion of a schema, where information that is aligned with knowledge and expectations can be easier to memorise (Alba & Hasher, 1983; Ghosh & Gilboa, 2014). Schemas have been hypothesised to encourage other mnemonic processes, for example by directing attention towards important aspects of stimuli (Alba & Hasher, 1983) or by enriching the representation and processing of stimuli (Miller, 2003).

In many circumstances, individuals use schematic knowledge to support memory, for example, by using a loved one’s name as a password or by using a meaningful date as a pin number. Empirical studies have shown that familiar material is easier to learn; for example, native words are easier to memorise than foreign words and nonwords (Hulme, Maughan, & Brown, 1991), native proverbs are easier to memorise than foreign proverbs (Poppenk, Kohler, & Moscovitch, 2010), and common visual scenes are easier to memorise than uncommon visual scenes (Badham, Hay, Foxon, Kaur, & Maylor, 2016). This also extends to individual differences in familiarity; for example, expert chess players are better able to visually memorise chess positions than novice players (Chase & Simon, 1973), and individuals more experienced with a given topic have been shown to be more able to memorise text based on that topic than less experienced individuals (Arbuckle, Vanderleck, Harsany, & Lapidus, 1990; Miller, 2003).

Early research by Bartlett (1932) postulated memory as a reconstructive process where schematic information was used to fill in gaps during recall. His work showed that after studying an unfamiliar folk tale, participants’ recall attempts often included simplification and distortions which aligned the details of the story with their own knowledge and culture. Modern research has built upon the work of Bartlett using his serial reproduction technique where the recalled information from one participant is presented as the study material for the next participant and so on. Over generations of serial reproduction, cultural studies have shown systematic shifts in the content of information, indicating that participants are imparting their own meaning onto study materials (Bangerter, 2000; Mesoudi & Whiten, 2004). Studies of language acquisition have explored serial reproduction with novel material in order to understand inductive biases in language learning. A key feature of such work is the fact that later generations of serial learners show more regularity in the material they report, demonstrating that individuals are imparting structure upon the information that they are learning (Reali & Griffiths, 2009; Smith & Wonnacott, 2010). This indicates that schema-like organisational processes are present in novel language learning, and that such processes are not unique to paradigms where schemas/meanings are concrete and explicitly identifiable.

Serial reproduction has also been investigated with more abstract stimuli. Ravignani, Delgado, and Kirby (2016) asked participants to learn random rhythmic patterns and their recall data were presented to later participants as study material and so on. Across generations of learners, patterns became more structured and easier to learn. Kalish, Griffiths, and Lewandowsky (2007) trained participants to learn functions mapping a stimulus magnitude *x* to a response magnitude *y*. Participants were trained on linear and nonlinear functions mapping *x* to *y* as well as random mappings. Across generations of learners, linear functions emerged in participants’ responses regardless of the function learned by the first generation (see also, Ferdinand & Zuidema, 2008). This indicated that a bias systematically distorted participants’ memory towards a simple relationship between *x* and *y*. Another study showed that, across generations of serial reproduction, participants tended to learn novel categorical information with a bias towards simpler organisational structures (Griffiths, Christian, & Kalish, 2008). The influence of an inductive bias has also been experimentally manipulated. Xu and Griffiths (2010) trained participants to learn sizes of imaginary fish for a classification task. Following this, participants completed a perceptual task where images of the fish were very briefly presented on the screen and participants were required to reconstruct their size. The reconstructions were used for later generations of participants and, across generations, the reconstructed sizes converged towards whatever size that group of participants had learned in the initial classification task.

Whilst serial reproduction tasks have been utilised to identify and classify inductive biases, there has been less emphasis on investigating the mnemonic properties of reproduced stimuli. Given that serial reproduction biases often converge towards prior knowledge and experience, and that prior knowledge and experience is known to support memory reconstruction, the current study aimed to establish if recall attempts for a given random stimulus (reconstructions) can be more memorable than the original stimulus. Ravignani et al. (2016), discussed above, established that recall attempts of random rhythmic patterns were easier to learn than the original patterns and other studies have shown improved learning in later generations of language learners (e.g., Kirby, Cornish & Smith, 2008; see Brighton Kirby & Smith, 2005, for a review). These previous studies have used multiple generations of learners, so it may be the case that a survival-of-the-fittest mechanism biases learning where easy to learn material is successfully transferred between learners but material more difficult to learn is lost. Crucially, this effect could be driven by biases in encoding, not retrieval. The current study focusses on retrieval by observing mnemonic effects across just one generation of learners, and by utilising a memory test where retrieval distortions can be measured.

Furthermore, to maximise the amount of structure participants could impart to stimuli during reproduction, the initial study material in the current study was entirely random, this is aligned with existing serial reproduction studies using novel stimuli. However, by utilising a paradigm based on the Visual Patterns Test (Della Sala, Gray, Baddeley, Allamano, & Wilson, 1999), our stimuli were richer than the novel stimuli in previous studies of serial reproduction and could be incorrectly recalled in many ways, affording greater opportunity for observation of retrieval biases. We also included a difficulty manipulation (study-test delay) in order to establish if mnemonic effects of reproduction are greater when recall is harder.

## Section 1: Mnemonic Tests

### Experiment 1

#### Method

##### Design

Participants studied visual matrix patterns and their memory was tested via recall either immediately or after a 15 seconds delay. Some of the patterns were random and some were previous participants’ recalled (PPR) patterns (recall based on immediate tests). The design was therefore 2 (study pattern: random, PPR) × 2 (test time: instant, delayed).

##### Participants

Thirty-two participants (21 Female) took part in the experiment aged between 19 and 31 years (*M* = 26.3, *SD* = 3.5). They were recruited from the local community and received £5 online shopping vouchers for participation. The study was approved by Nottingham Trent University’s College Research Ethics Committee.

#### Materials

The experiment was run on a laptop using Eprime 2.0. Thirty-two grids of patterns were created using a 4 × 4 array of squares which were randomly populated with eight white and eight black squares. The complete 4 × 4 grid was square with a side length of 10 cm corresponding to approximately 10° of visual angle at a viewing distance of 55 cm. Thin 6-pixel lines between each of the 16 squares were visible to mark their positions.

Participants also studied grids of patterns which were the recalled stimuli from previous participants (PPR). During recall, participants were forced to select exactly eight white and eight black squares, so the recalled stimuli had the same density as the random stimuli. PPR data were taken only from trials when memory was tested immediately after encoding. Previously recalled patterns were taken from the participant immediately preceding the current participant (an additional 31-year-old female recalled random patterns which were shown to Participant 1 and her data were not included in the analyses).

##### Procedure

Each visual pattern was displayed for 1,500 ms. It was then followed by two masks showing a chequered pattern in both orientations (black on white and white on black) each for 500 ms. In the instant recall trials, participants then immediately recalled the patterns. In the delayed recall trials, participants were instructed to complete simple true/false maths questions (e.g., 9–2 = 6, respond true/false) for 15 seconds before recall.

During recall, an empty grid was displayed (16 white squares) in the same size and position as the study grid. Participants were required to click the empty squares with a mouse pointer to turn them black (once black, squares could be clicked again to turn them white). Participants were given as long as they wanted to recall the pattern before clicking a “done” button to record their input. If they did not have eight black and eight white squares, an on-screen prompt encouraged them to select more or less black squares, whichever was appropriate (e.g., “8 black squares should be selected (currently you have 7)”).

Participants completed 12 trials of instant recall and 12 trials of delayed recall, blocked with order counterbalanced (half of the participants completed instant then delayed, and half delayed then instant). Within each of these 12 trials, four trials were recalled patterns from previous participants and eight trials were random patterns. The previously recalled patterns were never based on the same random patterns that were studied by the current participant. Data from the eight instantly-recalled, random patterns were allocated to the next participant as previously recalled patterns, half of which were randomly assigned to either the instant or the delayed recall conditions.

#### Results

##### Data preparation

Recall accuracy was scored as the proportion of the 16 squares that were the same as the encoded image. For PPR patterns, data were only included if the previous participant did not score 100% accuracy for that pattern. This is because 100% accuracy would be the same as testing with the original random pattern that the previous participant studied. Accuracy was relatively high so resulted in the exclusion of 69% of trials.

##### Participants analysis

A 2 (study pattern: random, PPR) × 2 (test time: instant, delayed) repeated measures ANOVA was completed with the accuracy data for each participant (see Table 1, for descriptive statistics). Fifteen participants had data in every cell of the design. There was no main effect of study pattern, *F* < 1. Instantly recalled patterns were recalled significantly better than delayed recall patterns, *F*(1, 14) = 45.06, *MSE* = 0.01, *p* < .001, $\eta_{p}^{2}$ = .76. There was no interaction, *F* < 1.

**Table 1:** Mean (and SD) Recall Accuracy—Proportion Correct—for Participants Studying Random or Previous Participants’ Recall Data (PPR) Tested Either Instantly or Delayed

Test Time	Study Pattern			
	Random		PPR	
	*M*	*SD*	*M*	*SD*
Instant	.95	.06	.92	.08
Delayed	.73	.13	.76	.17

*Note*. PPR = previous participants’ recall.

##### Items analysis

**Figure 1:**
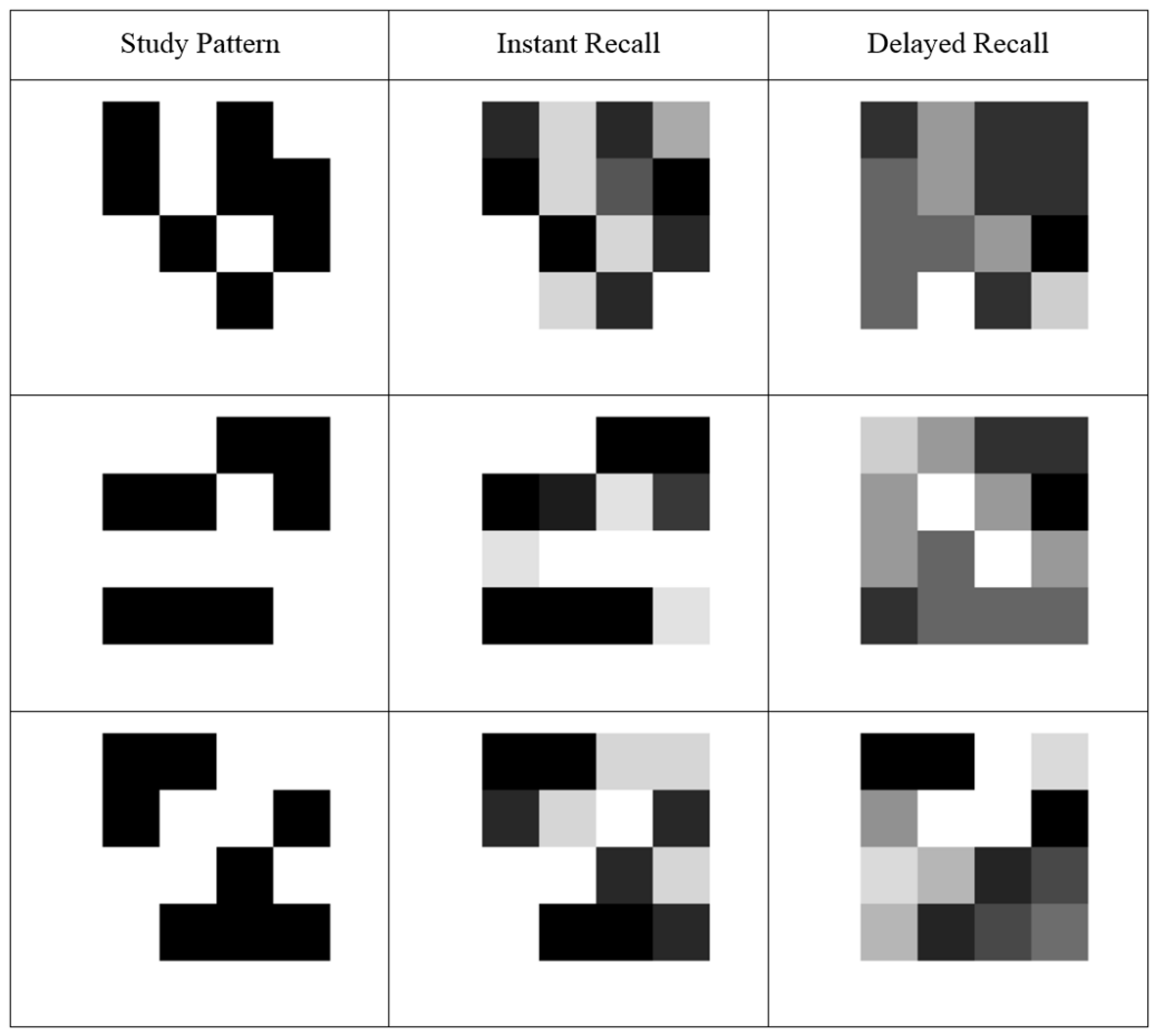
Mean accuracy (Experiment 1) for each element of three study patterns (left) depicted visually (percentage of participants recalling an element as black indicated from white 0% to black 100%) for instant (middle) and delayed (right) recall. *Note*. The supplemental materials show these data for all 32 study patterns used.

As there were 32 fixed random patterns, the same ANOVA as above could be calculated for the accuracy associated to each item (see Table 2, for descriptive statistics). Twenty one items had data in every cell. PPR data were recalled more accurately than random pattern data, *F*(1, 20) = 6.90, *MSE* = 0.02, *p* = .016, $\eta_{p}^{2}$ = .26, indicating that other individuals’ memory was more memorable than the original random source of that same information. As above, instantly recalled patterns were recalled significantly better than delayed recall patterns, *F*(1, 20) = 43.64, *MSE* = 0.01, *p* < .001, $\eta_{p}^{2}$ = .69. There was no interaction, *F* < 1. Additionally, the data allowed visualisation of how participants’ memory decayed between instant and delayed tests for each random image studied. Figure 1 shows examples of original study patterns and mean recall data for each element of the 4 × 4 study arrays.

**Table 2:** Mean (and SD) Recall Accuracy—Proportion Correct—for Each Item Displayed as Random or Previous Participants’ Recall data (PPR) Tested Either Instantly or Delayed

Test Time	Study Pattern			
	Random		PPR	
	*M*	*SD*	*M*	*SD*
Instant	.90	.07	.94	.10
Delayed	.67	.12	.76	.19

*Note*. PPR = previous participants’ recall.

#### Discussion

There was evidence in the data that memory reconstructions were more memorable than random stimuli in the items analysis but not in the participants analysis. Therefore, the overall result from Experiment 1 is inconclusive. A key issue was the selection of reconstructed patterns from previous participants for presentation to later participants. In many cases, the previous participant recalled patterns perfectly and these stimuli had to be removed from analyses as they were identical to random stimuli. This resulted in analyses based on a smaller pool of data when assessing the mnemonic properties of reconstructed patterns.

In Experiment 2, recall data from across all of Experiment 1 were used and this allowed us to deliberately select study patterns that were not recalled perfectly by earlier participants, resulting in no loss of data during analyses. Additionally, earlier participants’ recall data from both immediate and delayed recall tests in Experiment 1 were used as study material in Experiment 2. This was to further test the possibility that reconstruction biases may play a larger role when recall is more difficult (delayed recall), and more reconstruction is necessary (Alba & Hasher, 1983). Additionally, previous research using the Deese, Roediger, and McDermott procedure (Deese, 1959; Roediger & McDermott, 1995) has shown that false memory lures (memory distortions driven by schemas) are less susceptible to decay over time than original studied items (Seamon et al., 2002; Thapar & McDermott, 2001). It is hypothesised that schema-related information will persist throughout the delay better than non-schema-related information. Therefore, recall patterns produced after a delay may contain a greater proportion of schema-related material than instantly produced recall patterns, which may mean that delayed recall patterns are more memorable to later participants than instant recall patterns.

### Experiment 2

#### Method

##### Design

The design was the same as Experiment 1 except that half of the PPR patterns were now from trials where the previous participant recalled the pattern after a delay. The design was therefore 3 (study pattern; random, PPR-instant, PPR-delayed) × 2 (test time; instant, delayed).

##### Participants

Thirty six participants (20 female) took part in the experiment aged between 18 and 31 years (*M* = 24.9, *SD* = 3.7). None of the participants took part in Experiment 1. They were recruited from the local community and received online shopping vouchers for participation. The study was approved by Nottingham Trent University’s College Research Ethics Committee.

##### Materials

The study patterns were a subset of those used in Experiment 1. PPR data were taken from Experiment 1 as opposed to the participant immediately preceding the current participant. This allowed the selection of data for which recall accuracy was not 100% for a given pattern for both immediate and delayed recall—24 of the original 32 patterns were selected on this basis. Every non-100% recall attempt for a given pattern was used in the experiment pool; individual attempts were extracted from the pool at random for PPR trials. All 24 selected patterns were used with every participant, from these 24 patterns, eight were studied as original random patterns, eight were studied as PPR-instant, and eight were studied as PPR-delayed. From each set of eight, four were tested instantly and four were tested after a 15 seconds delay. Across participants, each pattern appeared equally in its original, PPR-instant or PPR-delayed forms and these forms appeared equally in instant and delayed recall trials. Instant and delayed recall trials were blocked and counterbalanced as in Experiment 1. Within the instant and delayed recall blocks, assigned patterns were presented in a random order.

##### Procedure

The second experiment tested visual pattern memory in immediate or delayed conditions identical to Experiment 1.

#### Results

##### Data preparation

Accuracy was defined in the same way as Experiment 1. Due to experimental error an anomalous pattern appeared for one trial for two participants before it could be corrected, and these two trials were excluded from the participants analysis. The corresponding item was excluded from the items analysis.

##### Participants analysis

A 3 (study pattern; random, PPR-instant, PPR-delayed) × 2 (memory test; instant, delayed) repeated measures ANOVA was completed with the accuracy data for each participant (see Table 3, for descriptive statistics). There was no main effect of study pattern, *F*(2, 70) = 1.56, *MSE* = 0.01, *p* = .219, $\eta_{p}^{2}$ = .04. Instantly recalled patterns were recalled significantly better than delayed recall patterns, *F*(1, 35) = 224.75, *MSE* = 0.01, *p* < .001, $\eta_{p}^{2}$ = .87. There was no interaction, *F* < 1.

**Table 3:** Mean (and SD) Recall Accuracy—Proportion Correct—for Participants Studying Random or Previous Participants’ Recall Data (PPR) Tested Either Instantly or Delayed

Test Time	Study Pattern					
	Random		PPR-Instant*		PPR-Delayed*	
	*M*	*SD*	*M*	*SD*	*M*	*SD*
Instant	.92	.09	.92	.09	.93	.09
Delayed	.70	.12	.75	.15	.74	.11

*Note*. PPR = previous participants’ recall.

*Previous participants’ recall from instant or delayed tests.

##### Items analysis

As for Experiment 1, items analysis was conducted using the same ANOVA structure as the participants analysis but with mean accuracy for each item as the dependant variable (see Table 4, for descriptive statistics). There was no main effect of study pattern, *F*(2, 44) = 1.12, *MSE* = 0.01, *p* = .335, $\eta_{p}^{2}$ = .05. Instantly recalled patterns were recalled significantly better than delayed recall patterns, *F*(1, 22) = 284.07, *MSE* = 0.01, *p* < .001, $\eta_{p}^{2}$ = .93. There was no interaction, *F*(2, 44) = 1.42, *MSE* = 0.01, *p* = .252, $\eta_{p}^{2}$ = .06.

**Table 4:** Mean (and SD) Recall Accuracy—Proportion Correct—for Each Item Displayed as Random or Previous Participants’ Recall Data (PPR) Tested Either Instantly or Delayed

Test Time	Study Pattern					
	Random		PPR-Instant*		PPR-Delayed*	
	*M*	*SD*	*M*	*SD*	*M*	*SD*
Instant	.92	.06	.93	.05	.93	.07
Delayed	.69	.11	.74	.10	.74	.09

*Note*. PPR = previous participants’ recall.

*Previous participants’ recall from instant or delayed tests.

### Discussion

The data showed no mnemonic benefits for study patterns constructed from previous participants’ recall attempts relative to random study patterns. This was the case for items and participants analyses. Given that a larger set of data was available for analyses in this experiment than in Experiment 1, the significant effect of study-pattern type for items analysis in Experiment 1 may be a Type I error (*p* = .016), with enhanced recall of previously studied patterns compared to random patterns driven by noise rather than systematic processes. Alternatively, in Experiment 1 there may have been a small subset of participants who showed mnemonic benefits for previous participants’ recall patterns across all items, but it is difficult to speculate what may have driven such an effect for some participants but not others.

One reason that mnemonic benefits were not found could be that there were not enough iterations of serial learning for participants to impart meaning onto the stimuli, although we deliberately avoided this for reasons mentioned in the introduction. Other studies that showed improved learning of earlier participants’ responses used several generations of learners (Kirby et al., 2008; Ravignani et al., 2016). Those studies focused on inductive biases and their stimuli were deliberately chosen to have the potential for evolution of structure. With the Visual Patterns Test, there are some patterns that are more learnable than others (Brown, Forbes, & McConnell, 2006) and it is likely that using multiple generations of learners would lead to memorable parts of the patterns remaining across generations and forgettable parts of the patterns changing across generations. A visual matrix test paradigm with multiple generations of learners might converge upon memorable patterns (such as the shapes of letters, cf. Brown et al., 2006) but we would be unable to easily attribute this to biases in encoding (the survival-of-the-fittest mechanism) or biases in retrieval/reconstruction. Of greater interest to the current study is how memory errors differ from random noise. The following section analyses systematic similarity in the errors produced within and between participants.

## Section 2: Homogeneity Tests

In order to quantitatively assess recall biases, analyses were conducted to assess the similarity of participants’ responses to 1) the responses of other participants and 2) responses to other trials within each participant’s own data. For every trial, the recall data for a “comparison” trial was compared to all “alternative” trials where comparison and alternative trials were never based on the same study patterns. Similarity was therefore calculated comparing the comparison trial to 1) all alternative recall data from *other* participants only or 2) all alternative recall data from that *same* participant only. As a baseline, the same comparisons were made but instead of comparing the comparison trial to alternative recall data it was compared to the alternative original studied patterns. Therefore, the only differences between the initial similarity measures (1 and 2) and their corresponding baseline measures were the errors made during recall. This was done to establish if the similarity measures were above baseline, which would indicate homogeneity of recall errors in the data.

Similarity was calculated following Nosofsky (1984). Each element of the recall data from the comparison trial was coded as a vector (*t*) of 16 ones and zeros representing the recall of black or white sections of a given 4 × 4 pattern. A corresponding vector (*x*) was created for each alternative trial to which the comparison trial was compared. The similarity between these two vectors was computed using the following equation:

$$Similarity\left(t,x\right)=e^{-c{\left(\overset{n}{\underset{j=1}{\sum }}{\left|t_{j}-x_{j}\right|}^{r}\right)}^{1/r}}$$ 1

where *j* cycles through each element of the vectors. For the current purposes, *r* was set to two to give an Euclidian distance metric (Nosofsky, 1984) and *c* was set to one. The average of these similarity measures was computed for the measures outlined above for 1) all comparisons to other participants 2) all comparisons to other trials from the same participant. The two baseline average similarities corresponding to (1) and (2) were computed based on the exact same trials but using the original study patterns instead of recall data in alternative trials. All of these four measures were computed separately using just instant recall trials or just delayed recall trials resulting in a 2 × 2 × 2 design (see below).

**Figure 2:**
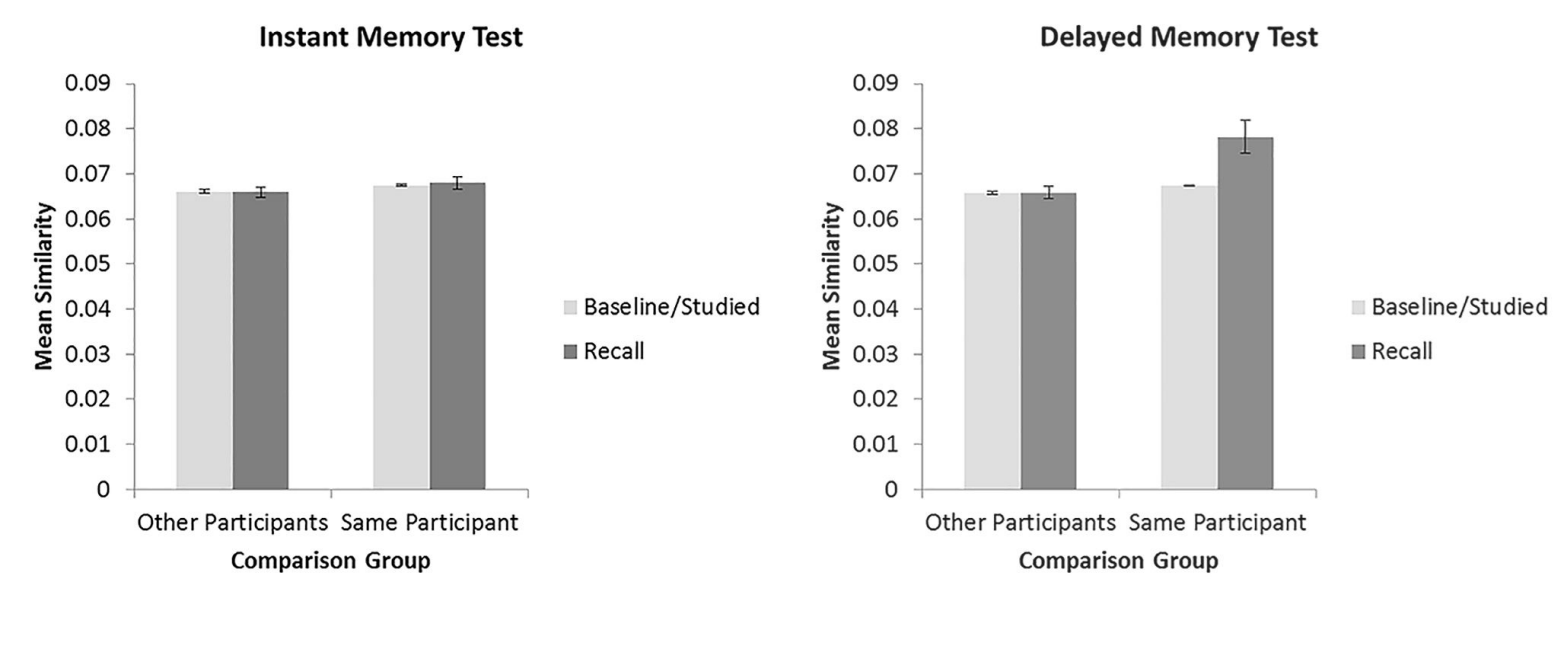
Mean similarity between trials from the same participant or between one participant to other participants for similarity to recall data or similarity to baseline/studied data. *Note*. Error bars are ± 1 *SE*.

As the above measures were computed for random study patterns only, Experiments 1 and 2 were comparable and were entered into the same statistical analysis. A 2 (comparison group; other participants, same participant) × 2 (comparison type; recall data, baseline/studied data) × 2 (memory test; instant, delayed) repeated measures ANOVA was computed on the mean summed similarity measures for each participant (see Figure 2, for descriptive statistics).^1^ A main effect of comparison group indicated that similarity was higher between data from the same participant than between a given participant’s data and other participants’ data, *F*(1, 67) = 14.17, *MSE* = 1.82 × 10^-4^, *p* < .001, $\eta_{p}^{2}$ = .18. A main effect of comparison type indicated homogeneity across recall responses—similarity was higher for comparisons between recall data than for comparisons of recall data to baseline/studied data, *F*(1, 67) = 11.48, *MSE* = 9.45 × 10^-5^, *p* = .001, $\eta_{p}^{2}$ = .15. There was no main effect of memory test, *F*(1, 67) = 2.76, *MSE* = 2.83 × 10^-4^, *p* = .102, $\eta_{p}^{2}$ = .04. Interestingly, an interaction between comparison group and comparison type revealed larger differences between recall data and baseline/studied data similarity measures for data from the same participant than for data from other participants, *F*(1, 67) = 12.85, *MSE* = 8.63 × 10^-5^, *p* = .001, $\eta_{p}^{2}$ = .16. This indicated homogeneity within the participants’ responses (similarity in excess of baseline similarity) was greater than homogeneity across different participants’ responses. Generally, homogeneity of responses only occurred for data based on delayed tests for comparisons within a single participant’s data (see Figure 2): There was an interaction between comparison group and memory test, *F*(1, 67) = 3.99, *MSE* = 2.38 × 10^-4^, *p* = .0497, $\eta_{p}^{2}$ = .06, between comparison type and memory test, *F*(1, 67) = 10.33, *MSE* = 9.24 × 10^-5^, *p* = .002, $\eta_{p}^{2}$ = .13, and a three-way interaction between all factors, *F*(1, 67) = 10.45, *MSE* = 8.23 × 10^-5^, *p* = .002, $\eta_{p}^{2}$ = .14. This pattern was confirmed by follow up paired *t*-tests across the comparison-type factor (comparing the mean similarity measures based on recall data as alternative trials to similarity measures based on baseline/studied data as alternative trials). For the following four t-tests, a Bonferroni correction was utilised with alpha set to .0125 for determining significance. For similarity to other participants, neither instant, *t* < 1, or delayed, *t*(67) = 1.07, *p* = .289, trials showed similarity in excess of baseline similarity. For similarity to other trials from the same participant, instant tests showed no similarity in excess of the baseline similarity, *t*(67) = 1.04, *p* = .314, but for delayed tests, there was significantly more similarity between participant’s own recall data than between recall data and baseline similarity, *t*(67) = 3.42, *p* = .001.

The homogeneity of errors observed could be simply due to misremembering as opposed to there being an innate structure to memory errors as one possible reason for the homogeneity of errors observed could be intrusions. Although we never calculated similarity measures between two sets of data based on the same study pattern, if a participant’s recall was an intrusion then this could enhance similarity if that intrusion was compared to the pattern on which the intrusion was based. For example, if a comparison trial was “Pattern 1” then this would be compared to all other alternative patterns (Patterns 2–24) to establish mean similarity. Therefore, if the participant failed to recall Pattern 3 but instead recalled an intrusion of Pattern 1, then this intrusion would have high similarity to Pattern 1 and would increase the overall mean similarity based on Patterns 2–22. To account for this, a different baseline measure was computed.

In the earlier analyses, comparison trials were based on recall data. The homogeneity of errors observed could have been due to an innate bias in the errors produced or due to intrusions. If it was due to intrusions, we would see the same enhanced similarity if comparison trials were recall data or if comparison trials were original studied data. This is because an intrusion would show high similarity to a previous trial regardless of whether the similarity measurement was based on recall data or the pattern that was originally studied as both would resemble the intrusion. Therefore, by utilising comparison trials using original studied data, a new baseline measure is formed and homogeneity above this indicates homogeneity unlikely to be due to intrusions. The measures of similarity were recomputed using original studied data as comparison trials for delayed recall only and for comparisons within the same participant only (i.e., for the only data that showed homogeneity—see Figure 2). With the new comparison trials, there was no significant difference between similarity based on alternative recall data versus similarity based on alternative original study patterns, *t* < 1 (*M* = 0.067, *SD* = 0.011; *M* = 0.066, *SD* = 0.010, respectively). Therefore, there was no indication that similarity was enhanced by intrusions. Furthermore, enhanced similarity across recall data was still present for delayed trials within a participant’s own responses, when compared to the new baseline measure that accounted for intrusions, *t*(67) = 3.13, *p* = .003. This indicates that the homogeneity observed in the earlier analyses was not due to intrusions but was due to an innate bias in the errors made by each participant.

## General Discussion

Across two experiments testing memory for visual matrix patterns, participants recalled patterns that were either random or were previous participants’ attempts to recall random patterns. It was initially hypothesised that previous participants’ recall attempts would be more memorable than random patterns because their recall errors may be influenced by reconstruction biases in individuals, reflecting what people are better able to learn. Experiment 1 showed some evidence of this with significantly better recall for study patterns based on previous participants’ recall than for random patterns, but this only occurred with items analysis and not for participants analysis and the effect did not replicate in Experiment 2, which had a larger data set. Despite this, there was evidence of homogeneity in the errors made by participants; when they made recall errors, the errors were similar across different trials when measured within participants. Furthermore, homogeneity of errors occurred within but not between participants and it could not be explained by intrusions. Overall, it appears that participants do show a bias in the way they recall visual matrix patterns. However, the bias may be unique to each individual and may lead to the production of patterns that are not particularly memorable to other individuals.

One aspect of the current study that differs from previous research based on learning material generated by other participants is the use of visual patterns as stimuli. The majority of research focussing on iterated learning over generations of participants is focussed on investigating language development (e.g., Kirby, Griffiths, & Smith, 2014). This is of interest to researchers because the acquisition of a language is an iterative process outside the laboratory (Kirby et al., 2008) with languages being transmitted throughout generations of individuals. Therefore, the abstract visual stimuli utilised in the current study may not have evoked the utilisation of mnemonic mechanisms hypothesised to have evolved specifically for the acquisition of language (cf. Pinker, 1994).

In contrast to the above, some previous studies do show improved learning in later generations using non-verbal material (e.g., rhythms, Ravignani et al., 2016; drawings, Tamariz, & Kirby, 2015) but mnemonic benefits seem to take multiple generations to manifest. Tamariz and Kirby argued that memory processes result in material becoming more compressible over iterations of learners. For example, in their study abstract drawings began to resemble letters across generations, allowing individuals to reproduce the shapes using their knowledge of the symbols. We have every reason to expect that a multiple-generation version of the current study would yield similar results, especially given that visual matrix patterns have been shown to be more memorable when they could be represented verbally (Brown et al., 2006). Therefore, we believe that mnemonic effects may take multiple generations to manifest with visual stimuli, where random memory errors eventually converge on schema-consistent patterns that resist degradation across generations. Although the use of a single generation was a potential limitation of the current study, it did allow us to utilize a much larger set of stimuli than those usually employed in multiple generation studies and to explore a generation of iterative learning in more detail, such as the comparison between a single individual’s responses.

Given that there was similarity in the errors made within an individual’s set of responses in the current data, it is highly likely that they would show higher accuracy in a memory test based on their own responses from earlier trials than they would show in a memory test based on random data. This is because in the absence of accurate recall, retrieval attempts based on guessing would match what was actually studied for a memory test that included guessing from earlier trials. That is, there appears to be consistency in guessing in memory tests for simple abstract visual stimuli. This would not be easy to test for as it would be difficult to dissociate from the memory benefits provided by repeated exposure to similar stimuli (cf. Hintzman & Block, 1971). Despite this, the current data are partly aligned with the generation effect, where material that is produced by an individual is easier to retrieve later by that same individual than material that is passively studied (e.g., Bertsch, Pesta, Wiscott, & McDaniel, 2007; Slamecka & Graf, 1978). A typical example of the generation effect involves generating a word from a clue; for example, finding a synonym of the word *rapid* beginning with *f* to get the target word *fast*. In later memory tests such target words are recalled better in generation conditions than in control conditions where a different participant simply reads the word pair *rapid-fast* (Slamecka & Graf, 1978). One mechanism by which the generation effect is proposed to operate is through transfer-appropriate processing; where the act of generation resembles processes that take place during retrieval, aiding memory (Bertsch et al., 2007). It may be the case that in the absence of memory for simple abstract visual stimuli, participants guess the pattern at retrieval. Then, when they forget parts of a pattern from a later trial, they are inclined to guess in the same way. This could explain why we saw more homogeneity within participants than between participants as the guessing would be highly dependent on the initial trials which were randomised for different participants.

Even though our data showed significantly more homogeneity of errors within participants than between participants, at least one iterated learning study has found largely equivalent inductive biases when comparing within- and between- participants designs (Griffiths et al., 2008). Given this discrepancy and the ideas expressed above, we tentatively propose two mechanisms that could bias the way information is stored and retrieved across generations of learners: 1) a survival-of-the-fittest mechanism where across multiple generations of learners, information that is easier to encode is more likely to be successfully transmitted to the next generation and 2), reconstruction biases with systematic influences on guessing in the absence of memory. This second mechanism could operate similarly to the generation effect as described above or could simply be that some stimuli are easier to guess than others. For example, as summarised in the introduction, Kalish et al. (2007) found that across generations of learners different *x-y* functions ended up as positive linear functions—it is established that simple linear functions are easier to learn than other types of function (Brehmer, 1971), possibly because this is the relation participants are most likely to guess (Naylor & Clark, 1968). It is therefore important for future researchers aiming to understand inductive biases to dissociate 1) effects based on what is easier to encode from 2) influences on guessing in the absence of memory.

## Data Availability

Original study patterns, demographics data, and raw data are freely available (see the Supplementary Materials section).

## Notes

The following supplementary materials are available via the PsychArchives repository (Badham, Atkin, & Castro, 2019; https://doi.org/10.23668/psycharchives.2591):

- Column description and file information
- Original study patterns
- Experiment 1 random study pattern visualisation
- Experiment 1 demographics data
- Experiment 2 demographics data
- Experiment 1 raw data
- Experiment 2 raw data
